# Complete genome sequence of bacteriophage P26218 infecting *Rhodoferax* sp. strain IMCC26218

**DOI:** 10.1186/s40793-015-0090-1

**Published:** 2015-11-24

**Authors:** Kira Moon, Ilnam Kang, Suhyun Kim, Jang-Cheon Cho, Sang-Jong Kim

**Affiliations:** School of Biological Sciences, Seoul National University, Seoul, 151-747 Republic of Korea; Department of Biological Sciences, Inha University, Incheon, 402-751 Republic of Korea

**Keywords:** Bacteriophage, *Rhodoferax*, Freshwater, *Podoviridae*, Genome

## Abstract

Bacteriophage P26218 is a virus that thrives in freshwater and infects *Rhodoferax* sp. strain IMCC26218, both of which were isolated from Soyang Lake, Korea. The bacterial host, IMCC26218, belongs to the genus *Rhodoferax* and is closely related to *R. saidenbachensis*, with 98.7 % 16S rRNA gene sequence similarity. Bacteriophage P26218 has an icosahedral head structure with a diameter of ~52 nm and short tail of ~9 nm, which is a typical morphology of the *Podoviridae* family. Its complete dsDNA genome was 36,315 bp with 56.7 % G + C content. This is the first genome sequence reported for a lytic phage of the genus *Rhodoferax*.

**Electronic supplementary material:**

The online version of this article (doi:10.1186/s40793-015-0090-1) contains supplementary material, which is available to authorized users.

## Introduction

Bacteriophages, which are obligate parasites of bacterial cells, are the most abundant biological entities that can be found in all biospheres [1–3]. Considering the fact that phages heavily influence the bacterial community structure [4] and various biochemical cycles such as the carbon cycle [5], understanding the genetic potential and diversity of phages would be important in the study of microbial community dynamics. Due to the lack of a universal phylogenetic marker gene to help understand phage diversity, several studies have been reported that include a survey of entire phage populations via metagenomics, from various environments including seawater, hot springs, soil, and freshwater [6–8]. These viral metagenomic studies demonstrate the extremely diverse nature and novel genetic repertoire of viruses, but the limited number of phage genomes poses a challenge for interpretation of virome data. Difficulty in phage isolation and genome sequencing is simply due to the lack of available bacterial hosts, since many of bacteria in natural environments are yet to be cultured [2]. Therefore, isolation of phages infecting major groups of bacteria and unveiling their genomic information are required to provide detailed information about each phage and enable meaningful interpretation of virome data.

The class *Betaproteobacteria* is often the most abundant group in freshwater environments, though less abundant in marine environments [9, 10]. Metagenomic studies on several freshwater bacteria revealed that the family *Comamonadaceae*, arbitrarily named betI [9], is the most frequently found family [11] within this class. The genus *Rhodoferax* [12], belonging to the family *Comamonadaceae*, is found in diverse habitats including ditch water, activated sludge, Antarctic microbial mats, and water reservoirs [10, 12–14]. Additionally, this is one of the most abundant genera within the 16S rRNA gene database [15]. Therefore, understanding the ecology of the genus *Rhodoferax* and its lytic phage will contribute to the understanding of freshwater microbial dynamics and help in further freshwater phage genomic studies. To isolate bacteriophages infecting *Rhodoferax* spp., we successfully isolated phage P26218, which infects *Rhodoferax* sp. IMCC26218 and further details of its genome features and annotations are described below.

## Virus information

### Classification and features

A bacteriophage, designated P26218 that infects the bacterial strain IMCC26218 was isolated from Soyang Lake, located inland of Gangwon-do, Korea, in October 2014. A bacterial strain, IMCC26218, was also isolated from the same site, using standard dilution plating technique on R2A agar (Becton, Dickenson and Company, Franklin Lakes, NJ, USA) in April 2014. Based on a comparative 16S rRNA gene sequence analyses, strain IMCC26218 was found to belong to the genus *Rhodoferax* with 98.7 % sequence similarity to *R. saidenbachensis* ED16^T^. To screen a representative lytic phage infecting representatives of the class *Betaproteobacteria*, *Rhodoferax* sp. IMCC26218 was used as the bacterial host.

**Fig. 1:**
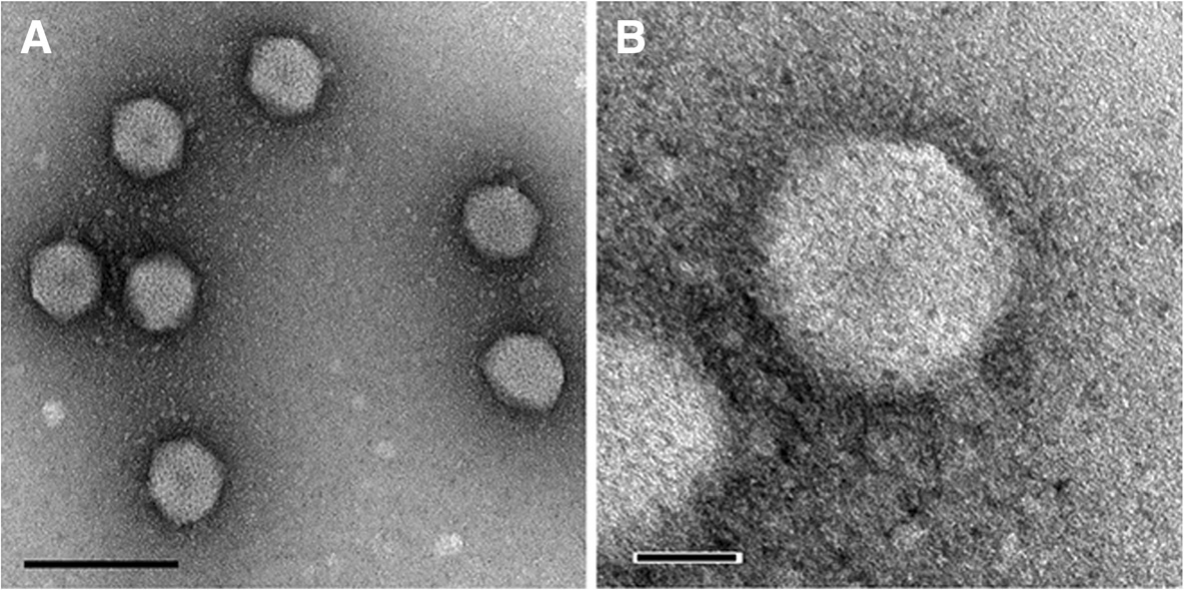
Transmission electron micrographs of phage P26218 particles infecting *Rhodoferax* sp. IMCC26218. The TEM images were obtained using Philips CM200 electron microscope. Scale bars represent 100 nm in (**a**) and 20 nm in (**b**)

**Fig. 2:**
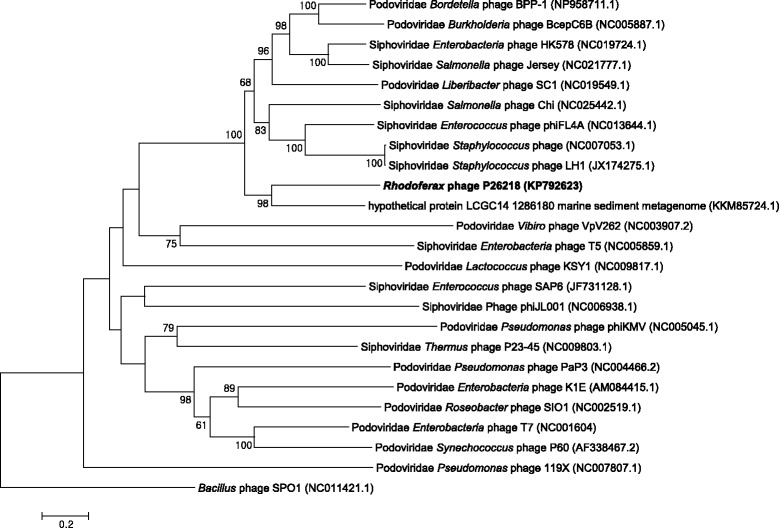
Phylogenetic tree highlighting the relationship of phage P26218 infecting *Rhodoferax* sp. IMCC26218 with representatives of the families *Podoviridae* and *Siphoviridae*. Sequences of DNA polymerase I (polA) collected from NCBI were aligned using CLUSTALW software [37], with *Bacillus* phage SPO1 (NC011421.1) as an outgroup. The phylogenetic tree was generated using the neighbor-joining method implemented in MEGA6 [38]. Bootstrap values representing over 60 % in 1,000 replicates are shown in the tree

Phage P26218 is a lytic phage that forms plaques of 1 to 2 mm in diameter, on *Rhodoferax* sp. IMCC26218 culture plates. Transmission electron microscopy of purified phage particles revealed its icosahedral-shaped head (52.1 nm in diameter) with a 9.4-nm long short tail (Fig. 1). The capsid encapsulates a linear dsDNA with length of 36,315 bp with 56.7 % G + C content. The morphology of the viral particle, including a uniform, icosahedral-shaped head with a short tail indicated that this phage belonged to the family *Podoviridae* of the order *Caudovirales* [16]. However, when its genomic characteristics were considered, no similar genomic architecture was found among the known viral genera, leaving P26218 without an assigned genus. The amino acid sequence of DNA polymerase I (encoded by polA) of P26218, one of the widely used viral phylogenetic markers [17, 18], was aligned with that of representative strains of the families *Podoviridae* and *Siphoviridae* and the aligned sequences were used for phylogenetic analysis. The phylogenetic tree based on DNA polymerase I revealed that P26218 formed a clade with a marine metagenome sequence, parted from previously known type species, confirming limitations in its assignment to a known genus (Fig. 2). A summary of the general phylogenetic features and isolation information are shown in Table 1.

**Table 1:** Classification and general features of phage P26218 infecting *Rhodoferax* sp. IMCC26218

MIGS ID	Property	Term	Evidence code^a^
	Classification	Genome group: dsDNA viruses, no RNA stage	IDA
		Phylum: unassigned	
		Class: unassigned	
		Order: *Caudovirales*	TAS [16]
		Family: *Podoviridae*	TAS [16]
		Genus: unassigned	
		Species: unassigned	
		Strain: P26218	
	Particle shape	Icosahedral head with a short tail	IDA
MIGS-6	Habitat	Freshwater lake, surface	IDA
MIGS-15	Biotic relationship	Intracellular parasite of *Rhodoferax* sp. IMCC26218	IDA
MIGS-14	Pathogenicity	Virulent phage of *Rhodoferax* sp. IMCC26218	IDA
MIGS-4	Geographic location	Soyang Lake, Gangwon-do, South Korea	IDA
MIGS-5	Sample collection	Oct. 17, 2014	IDA
MIGS-4.1	Latitude	37°57’11” N	IDA
MIGS-4.2	Longitude	127°49’02” E	IDA
MIGS-4.3	Depth	1 m	IDA
MIGS-4.4	Altitude		

^a^IDA: Inferred from Direct Assay and TAS: Traceable Author Statement. The evidence codes are from the Gene Ontology project [39]

## Genome sequencing information

### Genome project history

Compared to phage genomics and viromics in marine environments, relatively fewer studies with phage isolation and viral metagenome have been conducted in freshwater environments. Bacteriophage P26218 is the first lytic phage identified that infects the genus *Rhodoferax*, one of the representatives of the class *Betaproteobacteria* in freshwater environments. In this study, both virus and host were isolated from Soyang Lake in Korea. This phage was selected for genome sequencing as an initial approach to understand phages infecting members of the *Betaproteobacteria* isolated from surface freshwater in Korea. Genomic DNA was sequenced by the ChunLab Inc. Genome assembly, annotation, and submission to GenBank were performed at the Department of Biological Sciences, Inha University. This genome project was registered in Genomes Online Database, with accession ID, Gp0111341 as well as GenBank, with an accession ID of KP792623. A summary of the project information is described in Table 2.

**Table 2:** Project information

MIGS ID	Property	Term
MIGS 31	Finishing quality	Finished
	Number of contigs	1
MIGS-28	Libraries used	One paired-end Illumina library
MIGS 29	Sequencing platforms	Illumina Miseq
MIGS 31.2	Fold coverage	2,551×
MIGS 30	Assemblers	SPAdes version 3.1.1
MIGS 32	Gene calling method	RAST version 2.0, GeneMark.hmm version 3.25 and GLIMMER version 3.02
	GenBank ID	KP792623
	GenBank Date of Release	April, 2015
	GOLD ID	Gp0111341
	BIOPROJECT	NA^a^
MIGS 13	Source Material Identifier	NA^a^
	Project relevance	Diversity of freshwater bacteriophage

^a^Not available

### Growth conditions and genomic DNA preparation

The bacterial host, *Rhodoferax* sp. IMCC26218, was routinely cultured and maintained on R2A agar or in R2A broth (MB Cell, Los Angeles, CA, USA) at 20 °C. To screen lytic phages infecting this bacterial host, 10 l of water sample was collected from Soyang Lake at a depth of 1 m. The water sample was initially filtered using a 0.2-μm polyvinylidene difluoride membrane filter (Merck Millipore, Darmstadt, Germany) to remove bacterial-sized particles. To 400 ml filtrate, 100 ml of 5× R2A broth and 20 ml of IMCC26218 culture in the exponential phase were added, followed by incubation at 20 °C for 2 weeks for enrichment of bacteriophages that specifically infect *Rhodoferax* sp. IMCC26218. During the incubation period, 10 ml of the enrichment culture was sub-sampled 5 times at a 3-day interval. Each sub-sample was treated with approximately 3 ml chloroform to inactivate the bacterial cells. The treated samples were used for spot-double agar layer plaque assay on a *Rhodoferax* sp. IMCC26218 lawn plate for phage screening via appearance of plaques [19], resulting in the isolation of phageP26218.

The purification of phage P26218 genomic DNA was performed as per the ‘Molecular Cloning: A Laboratory Manual’ [20] with minor modifications. To 200 ml of phage lysate prepared for DNA purification, 1 μg ml^−1^ of DNase I and RNase A were added, followed by 11.7 g of NaCl. The obtained mixture was transferred to centrifuge bottles, to which PEG 8000 (Sigma-Aldrich, St. Louis, MO, USA) was added to attain a concentration of 10 % (w/v). After overnight incubation at 4 °C, the mixture was centrifuged at 11,000 × *g* for 40 min, supernatant was discarded by gentle inversion of the bottle, and the pellet was resuspended in 3–5 ml of SM buffer (50 mM Tris–HCl, pH 7.5; 100 mM NaCl; 10 mM MgSO_4_ · 7H_2_O; 0.01 % gelatin). PEG was removed from the liquid by treating it with equal volume of chloroform. The aqueous phase was then collected and further concentrated by ultracentrifugation at 246,000 × *g* for 2 h using a Beckman Coulter L-90 K ultracentrifuge with a SW 50 Ti swinging-bucket rotor. The phage pellet was resuspended in 100 μl SM buffer and used for genomic DNA extraction, using Qiagen DNeasy Blood and Tissue Kit, according to the manufacturer instructions.

### Genome sequencing and assembly

The genome of phage P26218 was sequenced at ChunLab Inc. using Illumina MiSeq system with 2 × 300-bp paired-end reads. The Illumina platform produced a total of 2 × 798,245 reads. The initial total reads were split by 2 × 50,000 reads into 16 sets [21] to facilitate the assembly process. Each set of sequence reads was independently assembled using SPAdes-3.1.1 [22], yielding a single contig but with different start points. Gap-closing PCR was performed with primers designed within the end region of a contig, which resulted in the circularization of the genome sequence. Circular assembly of the genome sequence suggested that the phage genome is terminally redundant or circularly permuted. This procedure for genome sequencing and assembly finally produced 36,315 bp with approximately 2,500× fold-coverage of the genome.

### Genome annotation

The ORFs were predicted using 3 gene prediction programs: GeneMark.hmm version 3.25 [23], Rapid Annotation using Subsystem Technology server version 2.0 [24], and NCBI Gene Locator and Interpolated Markov ModelER version 3.02 [25]. Only the ORFs that were identified by 2 of the 3 gene-prediction programs were included in the annotation. Each predicted ORF was translated and used to search for its homologous proteins and predict its domains using the NCBI BLASTP [17, 26], HHpred server [27] and HMMER [28] upon NCBI non-redundant database [26], the Conserved Domain Database [29], Pfam database [30], COG [31], PRK [29], and TIGRFam [32]. Then, TMHMM [33] and SignalP [34] were used to predict transmembrane helices and signal peptides.

## Genome properties

The properties and statistics of P26218 genome are summarized in Tables 3 and 4. The total length of the P26218 dsDNA genome was found to be 36,315 bp with 56.7 % G + C content. All 44 predicted ORFs were protein-coding sequences. However, only 15 of them were assigned to putative protein functions, while 29 were assigned to hypothetical proteins. One gene with a signal peptide was identified but none were found to have transmembrane helices.

**Table 3:** Nucleotide content and gene-count levels of the genome

Attribute	Value	% of Total^a^
Genome size (bp)	36,315	100.00
DNA coding (bp)	33,796	93.06
DNA G + C (bp)	20,589	56.70
DNA scaffolds	1	100.00
Total genes	44	100.00
Protein coding genes	44	100.00
RNA genes	0	0.00
Pseudo genes	0	0.00
Genes in internal clusters	0	0.00
Genes with function prediction	15	34.09
Genes assigned to COGs	11	25.00
Genes with Pfam domains	21	36.36
Genes with signal peptides	1	2.27
Genes with transmembrane helices	0	0.00
CRISPR repeats	0	0.00

^a^The total percentage is based on the total number of protein coding genes in the genome

**Table 4:** Number of genes associated with general COG functional categories

Code	Value	% of total^a^	Description
J	1	2.22	Translation, ribosomal structure and biogenesis
A	0		RNA processing and modification
K	1	2.22	Transcription
L	3	9.09	Replication, recombination and repair
B	0		Chromatin structure and dynamics
D	1	2.22	Cell cycle control, Cell division, chromosome partitioning
V	0		Defense mechanisms
T	0		Signal transduction mechanisms
M	0		Cell wall/membrane biogenesis
N	0		Cell motility
U	0		Intracellular trafficking and secretion
O	1	2.22	Posttranslational modification, protein turnover, chaperones
C	0		Energy production and conversion
G	0		Carbohydrate transport and metabolism
E	1	2.22	Amino acid transport and metabolism
F	2	4.44	Nucleotide transport and metabolism
H	0		Coenzyme transport and metabolism
I	0		Lipid transport and metabolism
P	0		Inorganic ion transport and metabolism
Q	0		Secondary metabolite biosynthesis, transport, and catabolism
R	2	4.44	General function prediction only
S	0		Function unknown
X	1	2.22	Mobilome: prophages, transposons
-	33	75.00	Not in COGs

^a^The percentage is based on the total number of protein-coding genes in the genome

## Insights from the genome sequence

**Fig. 3:**
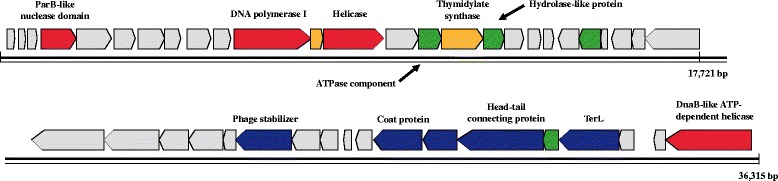
Genome map of *Rhodoferax* phage P26218. Total length of the genome is 36,315 bp and contig functions are color-coded as follows: light grey represents hypothetical proteins, yellow represents DNA metabolism, red represents DNA replication, blue represents structural and packaging genes and green represents other known functional genes

According to the genome annotation, bacteriophage P26218 is a unique phage, with no closely related phages. Therefore, this phage could only be classified based on its morphological characteristics, which attributed it to the family *Podoviridae*. Out of 44 predicted ORFs, only 15 (34 %) were assigned with a known function. As shown in Fig. 3, four ORFs were predicted to be related to DNA replication, 2 to DNA metabolism, 5 to packaging and structural functions, and 4 to other known functions (Additional file 1). BLASTP analyses showed that each ORF with an identified function was homologous to ORFs from different phages belonging to different viral families. All ORFs encoding viral packaging function were closely related to those of other viruses in the family *Podoviridae*. The ORFs encoding DNA polymerase I, ATPase component, thymidylate synthase, and hydrolase-like protein were similar to those of the family *Siphoviridae*, while the genes for DnaB-like ATP-dependent helicase and ParB-like nuclease domain showed a high degree of homology to those of the family *Myoviridae* This genomic architecture of P26218 confirmed the mosaic genome structure, known to be a result of lateral gene transfer usually predicted in viral genomes in attempts to enhance their genetic diversity [35, 36] and often observed in species of the order *Caudovirales* such as phages P22 and lambda.

## Conclusions

Lytic bacteriophage P26218, isolated from a freshwater lake is the first virus identified that infects the genus *Rhodoferax*. Based on its morphology, this phage was identified to be a member of the family *Podoviridae*, with an icosahedral-shaped head and short tail. All predicted ORFs from this phage genome were protein-coding, with 3 specifically coding for DNA replication, 7 for DNA metabolism, and 5 for packaging and structural proteins. The group of ORFs with similar function was postulated to originate from different groups of viral families (*Podoviridae*, *Siphoviridae*, and *Myoviridae*), which was indicative of the mosaic property of the P26218 genome. It is expected that phage P26218 isolated in this research and its genome sequence would be further used to study bacteria-phage interactions in freshwater environments, to reveal the evolutionary role of phage lateral gene transfer and to interpret freshwater virome data.

## Additional file

Additional file 1: Table S1. Description of data: Gene annotation table of bacteriophage P26218. (PDF 183 kb)

## Abbreviations

PEG: Polyethylene glycol
