# Supplementary material for: Complete genome sequence of bacteriophage P26218 infecting Rhodoferax sp. strain IMCC26218
Source: Stand Genomic Sci. 2015 Nov 24;10:111. doi: 10.1186/s40793-015-0090-1 (PMC4657236; doi:10.1186/s40793-015-0090-1)
Supplement: Additional file 1: Table S1. — Description of data: Gene annotation table of bacteriophage P26218. (PDF 183 kb) [file 40793_2015_90_MOESM1_ESM.pdf]

Supplementary Table 1. Bacteriophage P26218 gene annotation

**Gene**	Strand	Function or Similarity	Best BLASTP match (Accession no./ e-value/ virus family)[1]	Domain or Family (e-value)[2]
1	+	Hypothetical protein		
2	+	Hypothetical protein		
3	+	Hypothetical protein		
4	+	ParB-like nuclease	*Leptospira* phage LE1 (CAE14777.1/ 5E-04/ Myo)	TIGR04285 (8.10E-32)
5	+	Hypothetical protein		
6	+	Protein of unknown function	*Escherichia* phage N4 (YP_950527.1/ 9E-18/Podo)	PF11195 (3.10E-24)
7	+	Protein of unknown function	*Bacillus* phage PBC1 (YP_006383481.1/ 5E-17/ Sipho)	PF10926 (2.40E-38)
8	+	Protein of unknown function	Bacteriophage APSE-2 (ABA29383.1/ 4E-09/ Podo)	PF10926 (5.60E-46)
9	+	Protein of unknown function	*Clostridium* phage phiCT453A (AJA42507.1/ 1E-19/ UN)	PF10991 (1.80E-58)
10	+	Hypothetical protein		
11	+	PolA DNA polymerase I	*Staphylococcus* phage tp310-2 (YP_001429916.1/ 1E-96/ Sipho)	COG0749 (1.00E-101)
12	+	VRR-NUC domain	Bacteriophage APSE-5 (ACJ10148.1/ 8E-14/ UN)	PF08774 (3.10E-23)
13	+	Helicase	*Xylella* phage Xfas53 (YP_003344894.1/ 2E-103/ Podo)	COG0553 (2.1E-47)
14	+	Hypothetical protein	*Pseudomonas* phage vB_PaeS_PAO1_Ab18 (CEF89648.1/ 2E-22/ Sipho)	
15	+	Gln Q ABC-type polar amino acid transport system	*Pseudomonas* phage vB_PaeS_PAO1_Ab18 (CEF89651.1/ 6E-15/ Sipho)	COG1126 (7.60E-22)
16	+	Thymidylate synthase	*Pseudomonas* phage MP1412 (YP_006561023.1/ 8E-75/ Sipho)	PF00303 (4.70E-60)

17	+	HD containing hydrolase-like protein	*Pseudomonas* phage YuA (YP_001595841.1/ 6E-18/ Sipho)	PF12917 (4.70E-37)
18	+	Protein of unknown function	*Pseudomonas* phage M6 (YP_001294569.1/ 2E-15/ Sipho)	PF11753 (2.80E-26)
19	+	Hypothetical protein		
20	+	Hypothetical protein		
21	-	Hypothetical protein	*Thalassomonas* phage BA3 (YP_001552992.1/ 4E-15/ Podo)	
22	-	zliS Lysozyme family protein	*Acinetobacter* phage (YP_007010632.1/ 8E-63/ Podo)	COG3926 (5E-53)
23	-	Hypothetical protein		
24	-	Hypothetical protein		
25	-	Hypothetical protein		
26	-	Hypothetical protein	*Thalassomonas* phage BA3 (YP_001552270.1/ 4E-14/ Podo)	
27	-	Hypothetical protein		
28	-	Hypothetical protein	EBPR podovirus 1 (AEI70866.1/ 7E-30/ Podo)	
29	-	Phage protein	*Vibrio* phage VvAW1 (YP_007518345.1/ 1E-35/ Podo)	
30	-	Hypothetical protein	*Ralstonia* phage RSK1 (YP_008853798.1/ 2E-20/ Podo)	PHA00672 (2.80E-60)
31	-	Hypothetical protein		
32	-	Phage stabilisation protein	*Vibrio* phage VvAW1 (YP_007518349.1/ 3E-111/ Podo)	PF11134 (3E-148)
33	-	Hypothetical protein	EBPR podovirus 1 (AEI70872.1/ 6E-41/ Podo)	PF11650 (9.80E-12)
34	-	Hypothetical protein		

35	-	Hypothetical protein		
36	-	Hypothetical protein		
37	-	P22 Coat Protein	EBPR podovirus 1 (AEI70875.1/ 0/ Podo)	PF11651 (2.10E-99)
38	-	Phage-scaffold protein	EBPR podovirus 1 (AEI70876.1/ 2E-44/ Podo)	PF09306 (2E-25)
39	-	Head-tail connecting protein	EBPR podovirus 1 (AEI70877.1/ 0/ Podo)	PF12236 (2.90E-11)
40	-	GNAT acetyltransferase	EBPR podovirus 1 (AEI70878.1/ 1E-08/ Podo)	PF13718 (2.70E-15)
41	-	Phage terminase, large subunit	*Pelagibacter* phage HTVC010P (YP_007517700.1/ 3E-105/ Podo)	PF04466 (1.90E-44)
42	-	Hypothetical protein		
43	-	Hypothetical protein		
44	-	DnaB-like ATP-dependent helicase	*Yersinia* phage PY100 (CAJ28484.1/ 4E-11/ Myo)	COG1066 (1.90E-44)

[1]Best BLASTP match from the NCBI nr database (e-value ≤ 10^{-4}). For virus families, Podo represents family *Podoviridae*, Sipho represents family *Siphoviridae*, Myo represents family *Myoviridae*, and UN represents an unknown family.

[2]From search results of COG, Pfam, TIGRFAM and PRK, the one with most significant e-value (≤10^{-4}) was presented.
